# *BorreliaBase*: a phylogeny-centered browser of *Borrelia* genomes

**DOI:** 10.1186/1471-2105-15-233

**Published:** 2014-07-03

**Authors:** Lia Di, Pedro E Pagan, Daniel Packer, Che L Martin, Saymon Akther, Girish Ramrattan, Emmanuel F Mongodin, Claire M Fraser, Steven E Schutzer, Benjamin J Luft, Sherwood R Casjens, Wei-Gang Qiu

**Affiliations:** 1 Department of Biological Sciences, Hunter College, The City University of New York, 10065 New York, NY, USA; 2 Department of Computer Science, Hunter College, The City University of New York, 10065 New York, NY, USA; 3 Department of Biology, The Graduate Center, City University of New York, 10016 New York, USA; 4 Center for Translational and Basic Research, Hunter College, The City University of New York, 10065 New York, NY, USA; 5 Institute for Genome Sciences, University of Maryland School of Medicine, 21201 Baltimore, MD, USA; 6 Department of Medicine, New Jersey Medical School, Rutgers, The State University of New Jersey, 07103 Newark, NJ, USA; 7 Department of Medicine, Health Science Center, Stony Brook University, 11794 Stony Brook, NY, USA; 8 Department of Pathology, Division of Molecular Cell Biology and Immunology, University of Utah School of Medicine, 84112 Salt Lake City, UT, USA; 9 Department of Biological Sciences, Hunter College of the City University of New York, 695 Park Avenue, 10065 New York, NY, USA

**Keywords:** Lyme disease, Vector-borne relapsing fever, Genome browser, Recombination, Population genomics

## Abstract

**Background:**

The bacterial genus *Borrelia* (phylum Spirochaetes) consists of two groups of pathogens represented respectively by *B. burgdorferi*, the agent of Lyme borreliosis, and *B. hermsii,* the agent of tick-borne relapsing fever. The number of publicly available *Borrelia* genomic sequences is growing rapidly with the discovery and sequencing of *Borrelia* strains worldwide. There is however a lack of dedicated online databases to facilitate comparative analyses of *Borrelia* genomes.

**Description:**

We have developed *BorreliaBase*, an online database for comparative browsing of *Borrelia* genomes. The database is currently populated with sequences from 35 genomes of eight Lyme-borreliosis (LB) group *Borrelia* species and 7 Relapsing-fever (RF) group *Borrelia* species. Distinct from genome repositories and aggregator databases, *BorreliaBase* serves manually curated comparative-genomic data including genome-based phylogeny, genome synteny, and sequence alignments of orthologous genes and intergenic spacers.

**Conclusions:**

With a genome phylogeny at its center, *BorreliaBase* allows online identification of hypervariable lipoprotein genes, potential regulatory elements, and recombination footprints by providing evolution-based expectations of sequence variability at each genomic locus. The phylo-centric design of *BorreliaBase* (http://borreliabase.org) is a novel model for interactive browsing and comparative analysis of bacterial genomes online.

## Background

Spirochetes belonging to the genus *Borrelia* are obligate, tick- or louse-borne parasites of vertebrates and cause zoonotic infections in humans worldwide [1]. Phylogeny based on multiple genomic loci has confirmed the presence of two major *Borrelia* lineages, one represented by *B. burgdorferi*, the agent of Lyme borreliosis (LB), and the other by *B. hermsii*, the agent of tick-borne relapsing fever (RF) [2]. Members of the *Borrelia* appear to have the most complex genomic architecture among known prokaryotes. Sequencing of the first *Borrelia* genome revealed a 910-kilobase linear chromosome and twenty-one linear and circular plasmids with a total length of 610 kilobases in *B. burgdorferi* B31, a tick isolate from New York, USA [3,4]. Subsequent sequencing efforts showed that the linear chromosome is conserved and co-linear between the LB and RF groups [5-13]. A group of 30-kilobase circular plasmids appear to be conserved and syntenic across the entire genus [14]. A large ~160-kilobase linear plasmid specific to the RF group shares considerable syntenic regions with the lp54 plasmids of the LB group due to lateral transfer [15]*.* The linear plasmid lp23 in two RF group genomes is syntenic to the cp26 plasmids in LB group genomes [6]. Within the *B. burgdorferi* sensu lato species group (the LB group), a linear plasmid lp54 and a circular plasmid cp26 are universally present and largely syntenic among all species while the presence and the gene order of other plasmids are much less conserved [16,17]. Nevertheless, the total gene complement is quite stable within the LB group *Borrelia*[16,18].

The number of sequenced *Borrelia* genomes is rising rapidly. At the time of writing, the Bacterial Bioinformatics Resource Center (the PATRIC database, http://wwww.patricbrc.org) lists thirty-two completed and draft *Borrelia* genomes [19]. These genomic sequences represent twenty-eight LB *Borrelia* strains and six RF *Borrelia* strains, covering eight recognized or proposed species of the LB Group including *B. burgdorferi* sensu stricto, *B. afzelii*, *B. garinii*, *B. bavariensis*, *B. spielmanii*, *B. valaisiana*, *B. bissettii*, and *B. finlandensis*, and five species of the RF group including *B. recurrentis*, *B. duttonii*, *B. crocidurae*, *B. hermsii*, and *B. turicatae*. The chromosome of *B. miyamotoi*, an RF species, has also recently been sequenced [7]. A search on the GOLD genome-project registry (http://genomesonline.org/) yielded 47 completed, in-progress, and targeted *Borrelia* genome projects [20].

The rapid increase of sequenced *Borrelia* genomes allows for understanding *Borrelia* genome functions through comparative analysis, such as identification of host-interacting genes, regulatory elements, and pathways that are conserved among all species as well as species- and strain-specific genomic variations [21]. The comparative analysis of *Borrelia* genomes is presently hindered by a lack of dedicated online portal for their archival, dissemination, and comparative analysis*.* The Spirochete Genome Browser (SGD, http://sgb.fli-leibniz.de) currently provides web-based tools for analyzing the genomes of four strains of LB-group *Borrelia* (PKo, ZS7, B31, and PBi)*,* five strains of RF-group *Borrelia* (DAH, A1, 91E135, and Ly), three species of *Leptospiro* (*L. biflexa*, *L. borgpetersenii*, and *L. interrogans*), and two species of *Treponema* (*T. deticola* and *T. pallidum*). Web portals devoted to the comparative analysis of a single bacterial genus or species have been developed. A notable example is the *Pseudomonas* Genome Database, which facilitates population and evolutionary analysis of *Pseudomonas* genomes by providing up-to-date, computer-predicted orthologous gene sequences at both within- and between-species levels [22]. GenoList, a comprehensive database of over 700 completed bacterial genomes, provides tools and information for comparative proteomics, such as identification of proteins specific to a bacterial group [23]. At present, GenoList hosts genomic and proteomic information of three major *Borrelia* species causing Lyme disease (*B. burgdorferi* sensu stricto, *B. garinii*, and *B. afzelii*) and three *Borrelia* species causing relapsing fever (*B. recurrentis*, *B. duttonii*, and *B. hermsii*). The PATRIC database hosts a more comprehensive list of genomes of pathogenic bacterial genera including 34 genomes of *Borrelia*[19]*.* Comparative genomic information is also available from OrthologueDB, another comprehensive database of orthologous genes, which uses a rigorous, phylogeny-based algorithm to improve predictions of orthology in completed bacterial and archaeal genomes [24]. To the best of our knowledge, at present there is no genome database dedicated specifically to *Borrelia* or to providing comparative information such as sequences of orthologous ORFs in this genus.

We have recently released complete and draft genome sequences of twenty-one additional LB Group strains [10-13]. Population genomic analysis revealed genome-wide recombination among co-existing strains and adaptive genome diversification driven by antigenic variations within local *Borrelia* populations [17,25]. Despite large variations in the plasmid composition and the gene order on the majority of plasmids, the total gene content is remarkably stable among LB-group genomes, suggesting that species divergence is driven mainly by copy-number and adaptive sequence variations of surface lipoprotein genes [16]. Here we describe the design, content, and usage of *BorreliaBase*, a database developed during the course of the aforementioned studies. *BorreliaBase* is not only the first publicly available database dedicated to *Borrelia* genomes, it also implements a novel graphic user-interface design that encourages comparisons of bacterial genomes under a framework of their shared phylogenetic history.

## Construction and content

### *Borrelia* genomes

*BorreliaBase* is currently populated with sequences of 35 genomes representing eight LB-group *Borrelia* species, including *B. burgdorferi* sensu stricto*, B. garinii, B. afzelii, B. bavariensis, B. spielmanii, B. valaisiana, B. bissettii,* and *B. finlandensis*, and seven RF-group *Borrelia* species, including *B. miyamotoi*, *B. hermsii*, *B. parkeri*, *B. turicatae*, *B. crocidurae*, *B. recurrentis*, and *B. duttonii. B. burgdorferi* sensu stricto, a major pathogenic species causing Lyme disease in North America and Europe, is represented by genomes of eleven North American and two European strains. Table 1 lists sequenced *Borrelia* genomes that are presently in the *BorreliaBase*.

**Table 1:** **Complete and draft *Borrelia *** **genomes**^***a***^

**Strain**	**Species**	**Geographic origin**	**Biological source**^***b***^	**Sequencing status**	**NCBI Accession**^***c***^	**Genome report**
**Lyme borreliosis (LB) Group (*B. burgdorferi *** **sensu lato)**						
B31	*Bb sensu stricto*	New York, US	*I. scapularis*	Complete	PRJNA57581	[3,4]
64b	*Bb sensu stricto*	New York, US	Human	Draft	PRJNA55067	[13]
ZS7	*Bb sensu stricto*	Germany	*I. ricinus*	Draft	PRJNA59429	[13]
JD1	*Bb sensu stricto*	Massachusetts, US	*I. scapularis*	Complete	PRJNA161197	[13]
CA-11.2A	*Bb sensu stricto*	California, US	*I. pacificus*	Draft	PRJNA55063	[13]
CA382	*Bb sensu stricto*	California, US	N.A.	Complete (chromosome only)	PRJNA214794	N.A.
CA8	*Bb sensu stricto*	California, US	N.A.	Draft	PRJNA201452	N.A.
N40	*Bb sensu stricto*	New York, US	*I. scapularis*	Complete	PRJNA161241	[13]
72a	*Bb sensu stricto*	New York, US	Human	Draft	PRJNA54937	[13]
156a	*Bb sensu stricto*	New York, US	Human	Draft	PRJNA54817	[13]
WI91-23	*Bb sensu stricto*	Wisconsin, US	Bird	Draft	PRJNA55061	[13]
118a	*Bb sensu stricto*	New York, US	Human	Draft	PRJNA54935	[13]
297	*Bb sensu stricto*	Connecticut, US	Human	Complete (plasmids only)	PRJNA178345	[13]
29805	*Bb sensu stricto*	Connecticut, US	*I. scapularis*	Draft	PRJNA55055	[13]
Bol26	*Bb sensu stricto*	Italy	*I. ricinus*	Draft	PRJNA54819	[13]
94a	*Bb sensu stricto*	New York, US	Human	Draft	PRJNA54933	[13]
SV1	*B. finlandensis*	Finland	*I. ricinus*	Draft	PRJNA55065	[11]
DN127	*B. bissettii*	California, US	*I. pacificus*	Draft	PRJNA71231	[12]
PKo	*B. afzelii*	Germany	Human	Draft	PRJNA159867	[10]
ACA-1	*B. afzelii*	Sweden	Human	Draft	PRJNA54821	[10]
PBi	*B. bavariensis*	Germany	Human	Complete (chromosome, cp26, and lp54 only)	PRJNA58125	[8]
PBr	*B. garinii*	Denmark	Human	Draft	PRJNA55059	[10]
Far04	*B. garinii*	Denmark	Bird	Draft	PRJNA55149	[10]
VS116	*B. valaisiana*	Switzerland	*I. ricinus*	Draft	PRJNA54823	[12]
A14S	*B. spielmani*	The Netherlands	*I. ricinus*	Draft	PRJNA55069	[12]
BgVir	*B. garinii*	Russia	*I. persulcatus*	Draft	PRJNA162165	[26]
NMJW1	*B. garinii*	China	*I. persulcatus*	Complete	PRJNA177081	[27]
HLJ01	*B. afzelii*	China	Human	Complete	PRJNA177930	[28]
**Relapsing fever (RF) Group**						
A1	*B. recurrentis*	Ethiopia	Human	Complete	PRJNA58793	[6]
Ly	*B. duttonii*	Tanzania	Human	Complete	PRJNA58791	[6]
Achema	*B. crocidurae*	Mauritania	*O. sonrai*	Draft	PRJNA162335	[5]
DAH	*B. hermsii*	Washington, US	Human	Draft	PRJNA59225	N.A.
HR1	*B. parkeri*	California, US	*O. parkeri*	Complete	PRJNA231102	[29]
91E135	*B. turicatae*	Texas, US	*O. turicatae*	Complete (chromosome only)	PRJNA58311	N.A.
LB-2001	*B. miyamotoi*	Northeast US	*I. scapularis*	Complete (chromosome only)	PRJNA215233	[7]

^*a*^Sources: PATRIC (http://wwww.patricbrc.org) and GOLD (http://genomesonline.org/), accessed on December 21, 2013. Sequencing status: “Complete”, gaps closed; “Draft”, gaps not closed. “N.A.”, information not available.

^*b*^*I. scapularis, I. ricinus, I. persulcatus*: hard-bodied *Ixodes* tick species. *O. sonrai, O. turicatae, O. parkeri*: soft-bodied *Ornithodoros* tick species.

^*c*^NCBI BioProject (http://www.ncbi.nlm.nih.gov/bioproject) RefSeq Genome Accession.

While *BorreliaBase* includes basic genome annotations such as plasmid identity, ORF descriptions, and graphic maps of ORF locations, single-genome information is not the focus of *BorreliaBase*, considering that such information is available in primary or aggregate databases such as the NCBI Microbial Genomes website (http://www.ncbi.nlm.nih.gov/genomes/MICROBES/microbial_taxtree.html), the UCSC Microbial Genome Browser (http://microbes.ucsc.edu/), and the PATRIC database (http://patricbrc.org). Instead, *BorreliaBase* focuses on providing high-quality comparative information for sequenced *Borrelia* genomes including genome phylogeny, genome synteny, and orthologous ORFs and intergenic spacers (IGSs) (Table 2).

**Table 2:** **Main features of *BorreliaBase***

**Comparative feature**	**Contents**	**Utility**
**Genome phylogeny**	• Based on 266 sets of single-copy orthologs present in all *Borrelia*	• Select or de-select genomes
	• Used as a navigation guide and a framework for genome comparison	• Generate phylogenetic expectations
**Plasmid composition**	• Length and identity of 489 contigs, inferred based on sequences of plasmid partition genes	• Identify presence and absence of plasmids
**Genome synteny**	• Gene co-linearity of lp54, cp26, and the main chromosome	• Identify gene gains and losses (Figure 2)
	• Across 35 genomes from 8 species of LB-group *Borrelia* and 7 species of RF-group *Borrelia*	
**Orthologous IGSs**	• 541, 29, and 17 orthologous IGS families from main chromosome, lp54, and cp26, respectively	• Identify conserved regulatory sequences through phylogenetic footprinting (Figure 3)
	• Start and end positions manually curated according to consensus start-codon positions of flanking orthologous ORFs	
**Orthologous ORFs**	• 750, 61, and 26 orthologous ORF families from main chromosome, lp54, and cp26, respectively	• Visualize synonymous and nonsynonymous variations (Figure 4)
	• 5’ end sequences of each ORF manually curated according to the consensus start-codon position of each ortholog ORF family	• Identify recombination through homoplasy (phylogenetic inconsistency) (Figure 5)

#### Orthologous ORFs and IGSs

At its current state, the *Borreliabase* contains sequences of 750, 26, and 61 orthologous ORF families on the main chromosome, the cp26 plasmid, and the lp54 plasmid, respectively [17], as well as sequences of 541, 17, and 29 sets of unpublished orthologous IGSs on the main chromosome, the cp26 plasmid, and the lp54 plasmid, respectively. Methods on the identification of orthologous ORFs on the main chromosome, lp54, and cp26 have been described previously [17]. Briefly, the ORF orthology was determined with a combination of sequence-similarity search using BLAST [30], clustering of homologous proteins using MCL [31], and manual validation based on genome synteny. Subsequently, we identified orthologous IGS sequences as those sharing a pair of flanking orthologous ORFs. To ensure the quality of IGS sequences in individual genomes, a consensus start-codon position was identified for each orthologous ORF family with the assumption that start-codon position and N-terminus sequences are conserved within as well as between species. As a result, many ORF sequences have been manually adjusted and their N-terminus sequences may differ from those in NCBI and other genome repositories. Plans have been made to reconcile ORF coordinates between *BorreliaBase* and the NCBI genome repository.

#### Genome-based phylogeny

**Figure 1:**
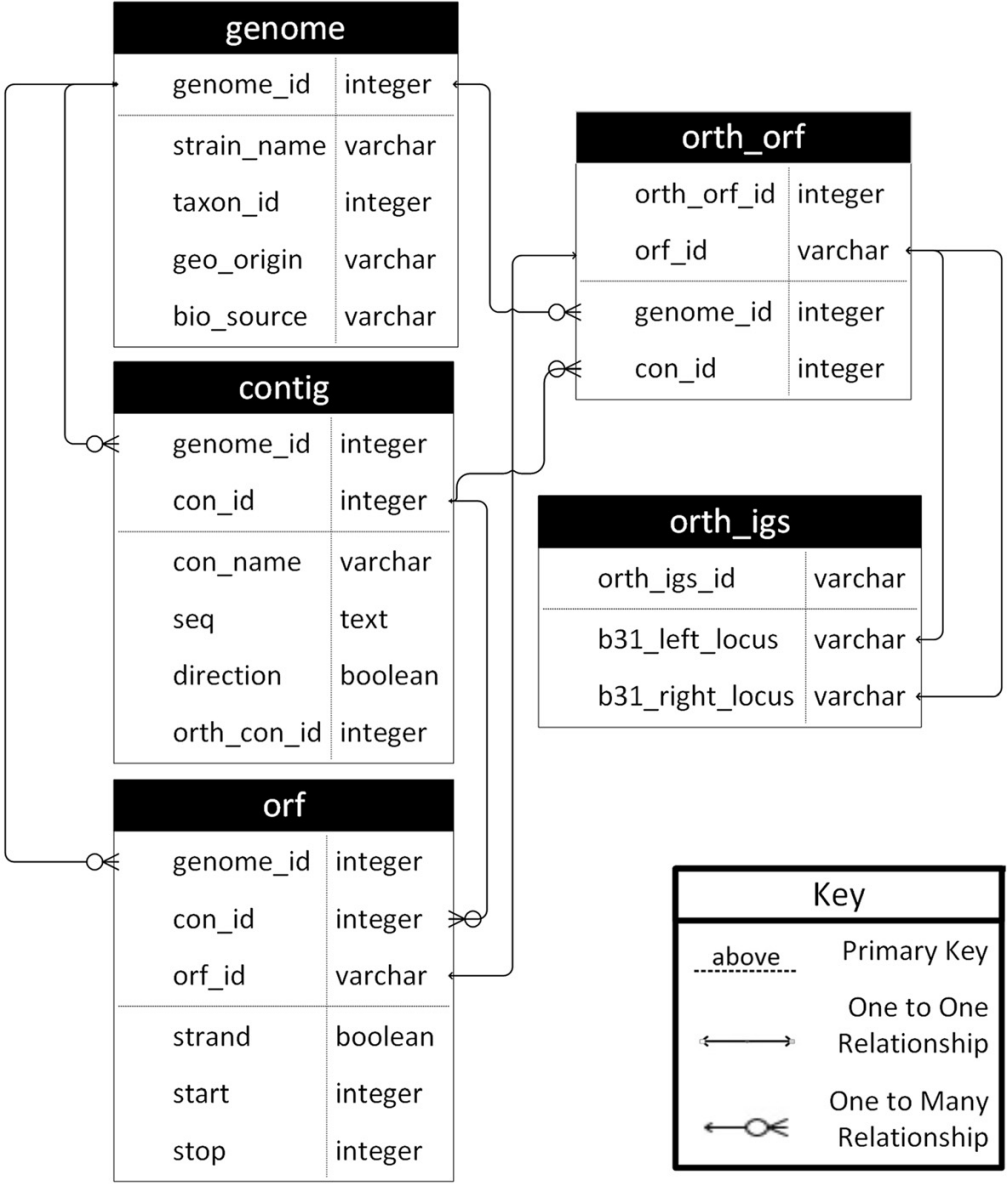
**Database schema.** Five main relational entities and their relations are shown. Each row of the “genome” table describes a sequenced genome. Each row of the “contig” table contains the nucleotide sequence of a plasmid (for completed genomes) or contig (for draft genomes). Each row of the “orf” table lists coordinates and coding direction of an ORF. Each row of the “orth_orf” the table assigns an ortholog family identifier (“orth_orf_id”) to an ORF. Each row of the “orth_igs” table defines a unique intergenic spacer (IGS).

Currently, comparative data on *BorreliaBase* include a statistically robust genome phylogeny. First, 266 sets of single-copy ortholog ORF families present in all 35 *Borrelia* genomes were identified from the ortholog database (Figure 1). Subsequently, each ORF family was translated into protein sequences, which were then aligned with MUSCLE (v3.7) [32]. Codon alignments corresponding to aligned protein sequences were obtained and then concatenated using customized PERL scripts based on BioPerl [33]. A maximum likelihood tree was obtained by using RAxML with three partitions of nucleotide substitution rates corresponding to the three codon positions [34]. Most of these orthologs are chromosome-borne. The phylogenetic position of the strain 297, the main chromosome of which remains to be sequenced, was determined and manually added to the RAxML tree based on phylogenies previously derived using plasmid-borne SNPs [18].

#### Sequence alignments

In addition to serving the raw orthologous sequences, *BorreliaBase* provides alignments of these sequences to allow direct interactive exploration of sequence variability (see Utility and Discussion). For orthologous IGS sequences, we provide online visualization of nucleotide alignments produced by MUSCLE (v3.7) with individual sequences ordered according to the genome phylogeny (see Use Case 3 below). For each set of orthologous ORF sequences, we provide MUSCLE-aligned amino-acid sequences as well as the corresponding codon alignment based on the amino-acid alignment (see Use Case 2 below).

### Relational database and web interface

*BorreliaBase* is supported by a database in the back end with five main relational entities (Figure 1). The database is implemented with the PostgreSQL database management system (server version 9.1.9, http://www.postgresql.org). A dynamic web front was developed using the Asynchronous HTTP Request (AJAX) technology. On the server side, customized Perl-based Common Gateway Interface (CGI) scripts receive user inputs, fetch sequences from the back-end database, perform alignment if necessary, and output requested sequences and alignments as JavaScript Object Notation (JSON) objects. On the client side, customized JavaScript functions built with the jQuery (http://jquery.com) and D3 (http://d3js.org) libraries render these JSON objects into dynamic HTML contents.

## Utility and discussion

With *BorreliaBase*, we aim to facilitate comparative analysis of *Borrelia* genomes in a phylogenetic framework. For example, users can download manually curated orthologous ORF and IGS sequences or directly visualize sequence variations online. We do not duplicate existing single-genome features already provided by general-purpose microbial genome browsers such as the NCBI Microbial Genomes and the Integrated Microbial Genomes (IMG) databases. These databases provide comprehensive genome annotations including genome maps, plasmid identities, and ORF descriptions. In the following, we describe four examples to illustrate how *BorreliaBase* may be productively used for comparative exploration of *Borrelia* genomes.

### Use case 1: synteny analysis- identify gene gains and losses

**Figure 2:**
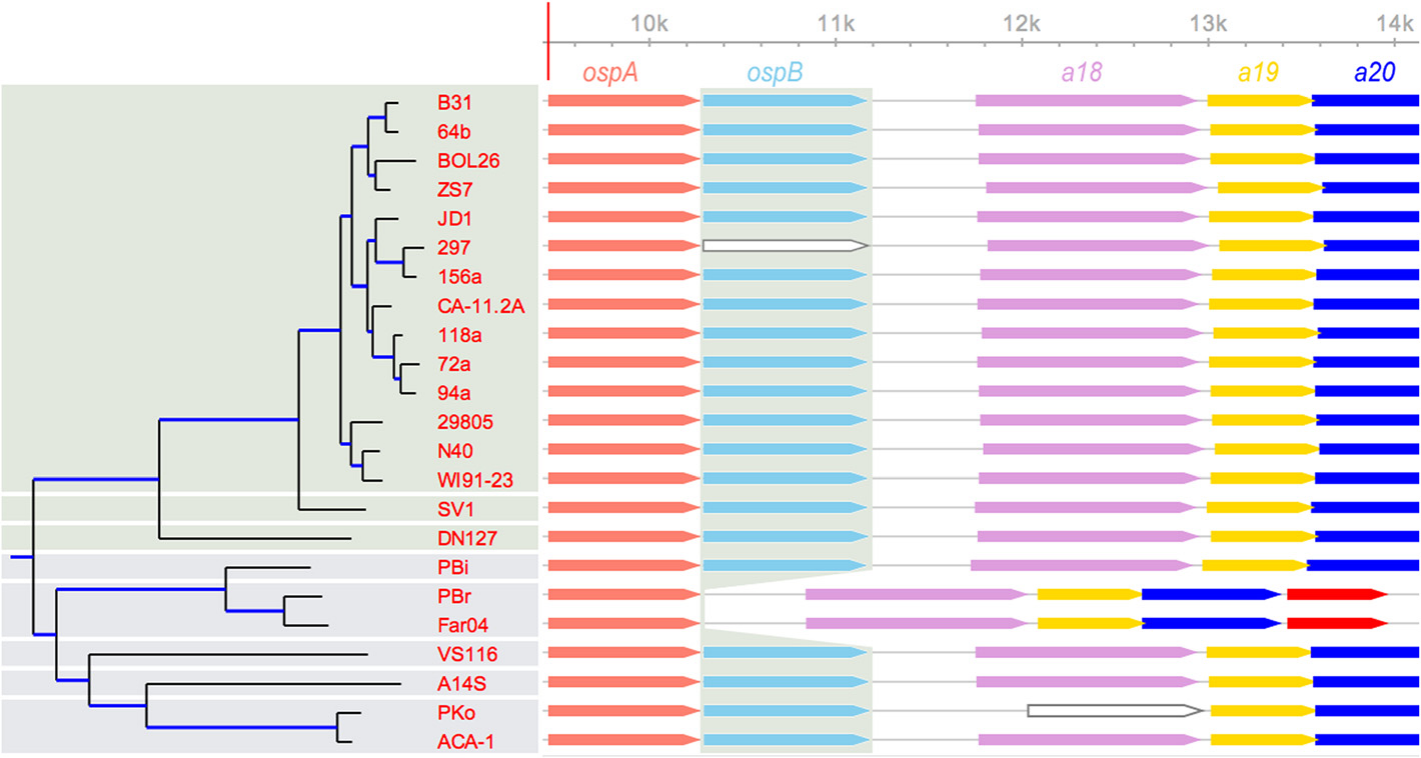
**Use case 1.** Identify gene gains and losses. In this synteny view of *BorreliaBase*, orthologous ORFs are shaded with the same colors (*right*) and arranged according to the genome phylogeny (*left*). Such a display helps to identify evolutionary gains and losses of genes. In this example, the gene *ospB* (shared vertically in gray) is apparently lost during the evolution of *B. garinii* (represented here by two strains PBr and Far04). Uncolored ORFs contain frameshift mutations, which may also be sequencing errors. The synteny view of *BorreliaBase* is dynamic rather than static, allowing users to obtain customized synteny views centered on any specific gene (*ospA* in this case, marked by a vertical red line on the ruler, which is based on the scale of the B31 genome).

Synteny analysis compares co-linearity of genes on a replicon to identify evolutionary gains and losses of genes in genomes. A reliable synteny analysis requires the identification of a set of orthologous genes across multiple genomes as well as a phylogeny of these genomes. In this example, it has been identified that orthologs of *ospB* is absent in the genomes of two *B. garinii* strains PBr and Far04 (Figure 2). By displaying gene synteny in the context of the genome phylogeny, *BorreliaBase* allows us to conclude based on the principle of maximal parsimony that there is a single, lineage-specific deletion of *ospB* on the branch leading to *B. garinii*. This evolutionary history is consistent with the function of *ospB* being a gene expressed primarily in a mammalian environment [35] and with the fact that *B. garinii* uses birds as primary reservoir hosts [36]. Since the synteny view on *BorreliaBase* serves dynamic rather than static images, one can obtained customized synteny views centered on a specific gene (*ospA* in this case, Figure 2). A further customization is that one may select or hide genomes by clicking on the branches or tips of the tree.

### Use case 2: phylogenomics- visualize synonymous and nonsynonymous variations

Lipoproteins, especially those localized to the outer cell membrane, are primary candidates for the development of diagnostics for and vaccines against *Borrelia* infection, since they play key roles in establishing infections in arthropod vectors and vertebrate hosts [37,38]. For example, *B. burgdorferi* undergoes phase changes in its outer surface protein composition through the expression of *ospA* in the tick phase, the expression of *ospC* during host invasion, and the expression of *vls* genes in the mammalian phase [39]. Genes encoding outer surface lipoproteins tend to show elevated sequence variability within species, between species, or both within and between species [16,40]. As such, sequence variability is a reliable guide for identifying *Borrelia* proteins and protein regions that interact directly with the host*.* A frequent request *Borrelia* researchers make to us is the identification of sequences orthologous to a gene of interest in all sequenced genomes. *BorreliaBase* fulfills this need by providing online access to orthologous ORF sequences.

**Figure 3:**
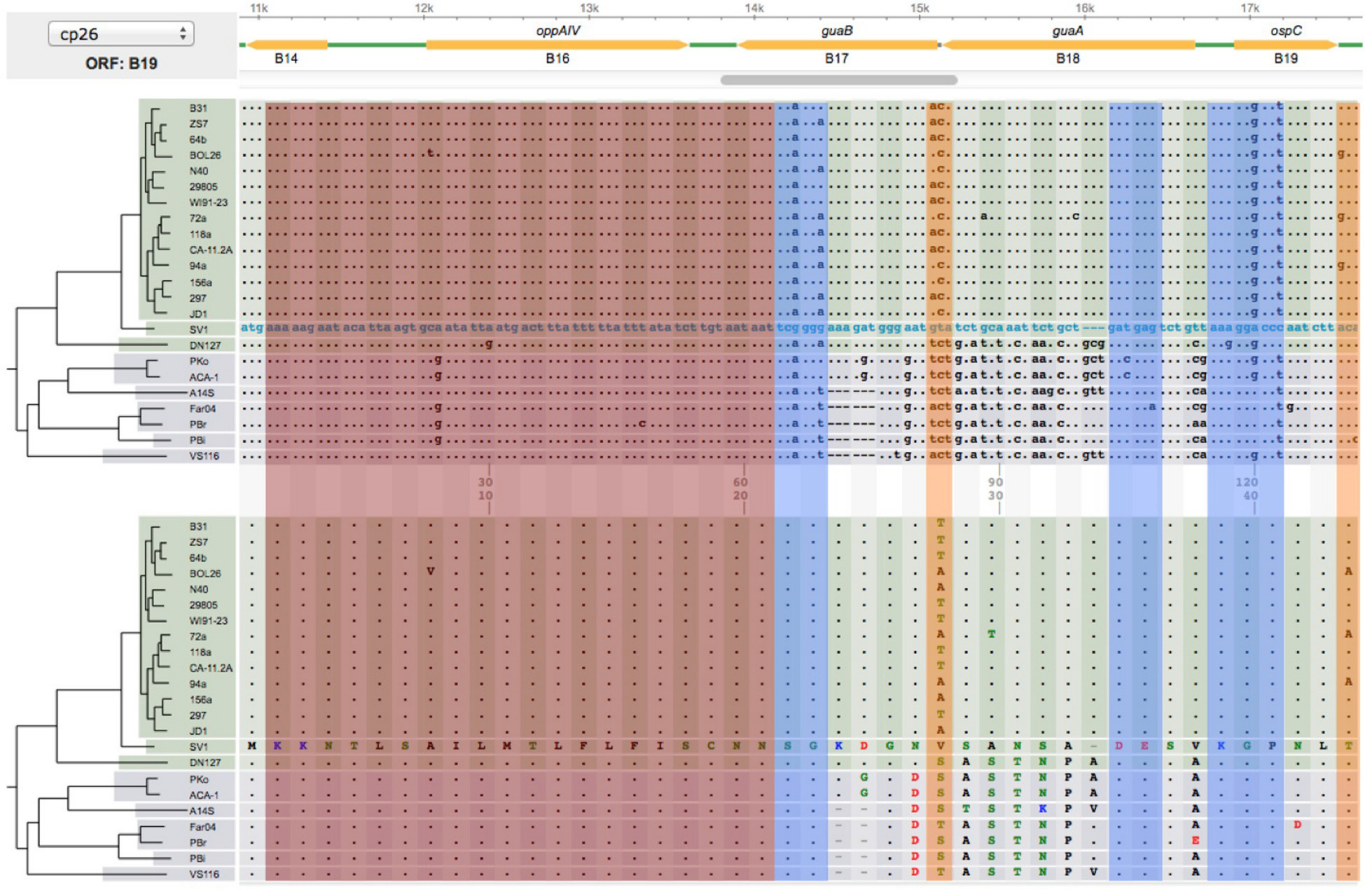
**Use case 2.** Identify synonymous and nonsynonymous variations. Integrated visualization of codon (top panel) and amino-acid (bottom panel, colored by chemical groups) alignments, with individual sequences arranged according to the genome phylogeny, allows identification of synonymous and nonsynonymous variations. This view also facilitates exploration of selective mechanisms underlining sequence variations. Here, three notable patterns of sequence variability are highlighted for the first 44 codons of *ospC*. Some sites vary synonymously within and between species (high-lighted in blue), indicating strong purifying selection. Two sites vary non-synonymously within a single species (in orange), suggesting balancing selection. The first 21 sites (in red) are nearly constant not only in amino acids but also in codons, hinting strong selection for codon usage in this gene region.

A user begins by searching for a gene of interest using either a locus name (e.g., a15, the locus name for *ospA* in strain B31, the reference genome for LB group *Borrelia*) or a gene name (e.g., *ospA*). If the gene exists, a synteny view centered on the requested ORF appears showing its genomic context (Figure 2). At the same time, both the amino-acid alignment and the corresponding codon alignment of its orthologs are displayed (Figure 3). The integration of codon and amino-acid alignments allows easy determination of whether a nucleotide substitution is synonymous or non-synonymous (Figure 3). With the codon-alignment view, a user is able to visually scan for genes or regions that are potentially host-interacting based on a high density of non-synonymous substitutions before conducting a formal nonsynonymous-to-synonymous (*K*_*A*_*/K*_*S*_) ratio test [41]. Conversely, a user may identify gene regions with both low rates of synonymous and nonsynonymous substitutions between the *Borrelia* species. For example, using the codon-alignment view of *BorreliaBase,* one can visualize that the 5’ leader sequences of *ospC* is highly conserved across the eight LB-group *Borrelia* species in both synonymous and nonsynonymous nucleotide sites (Figure 3). Conservation of synonymous sites at the 5’ regions of a gene is common in bacteria and is typically associated with a preference for rare codons and a high level of gene expression [42].

A user may download aligned and unaligned DNA and protein sequences of a set of orthologous ORFs. Further, users may select or de-select strains using the genome phylogeny.

### Use case 3: phylogenetic footprinting- identify regulatory sequences

**Figure 4:**
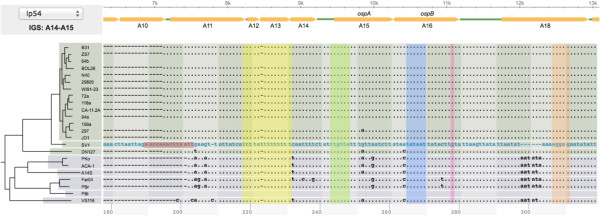
**Use case 3.** Identify conserved regulatory sequence. In this alignment view of the promoter region of the *ospA/ospB* operon, a dot (“.”) represents a nucleotide or a gap that is the same as in strain SV1, the closest outgroup of *B. burgdorferi* sensu stricto and used as the reference. Known *cis-*regulatory elements within this promoter region are highly conserved across LB-group *Borrelia* species, including, from left to right, a direct repeat (DR, in purple) and the T-rich region (in yellow) associated with RpoS-mediated repression in mammalian environment, the putative RpoD-binding sites (−35 region in green and −10 region in blue), the transcription start site (“+1”, in pink), and the ribosome-binding site (RBS, in orange).

For each set of orthologous IGS sequences, a user can browse a nucleotide alignment to identify potential *cis-*regulatory sequences by looking for nucleotide sites conserved among the *Borrelia* species. Functional elements embedded in IGSs, e.g., *cis-*regulatory sequences and sequences encoding small RNAs, tend to be conserved among closely related bacterial species, allowing identification of such elements through comparative genomics with an approach called phylogenetic footprinting [43-45]. Using *BorreliaBase*, for example, we were able to show a high level of cross-species conservation of *cis-*regulatory sequences upstream of the *ospAB* operon, including the ribosomal-binding site, the transcription start position, the −35 and −10 RpoD (σ^70^)-binding sites, and a T-rich region required for *ospA/B* repression in mammalian hosts [46] (Figure 4).

### Use case 4: population genomics- identify recombination

**Figure 5:**
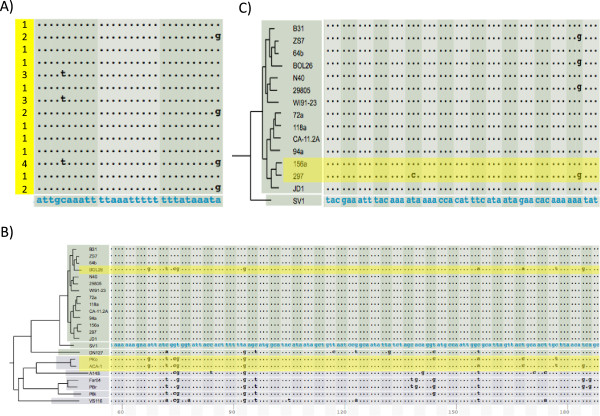
**Use case 4.** Identify recombination. Recombination among bacterial genomes leaves genomic footprints that are identifiable by tests of homoplasy (*i.e.,* phylogenetic inconsistency). **(A)** The “four-gametes” test. Two SNP sites within the *b18-b19* IGS region are shown. All four possible haplotypes (labeled “1” through “4” and highlighted in yellow) are present in this region among the 14 *B. burgdorferi* sensu stricto genomes, suggesting at least one recombination event between these two SNP sites. **(B)** Cross-species gene conversion. A codon alignment of a region of *b22* is shown. The *B. burgdorferi* sensu stricto strain BOL26 has a haplotype in this region that closely resembles its homologs in two *B. afzelii* strains (PKo and ACA-1), caused apparently by a replacement of the allele in BOL26 by an allele originated from a *B. afzelii* strain. **(C)** The “sister-group” test. Two SNP sites in *a18* segregating between 156a and 297, two phylogenetic sister-group strains, are shown. The “C” at the first SNP site is a singleton while both alleles at the second SNP site have multiple copies. Since sister-group genomes are most closely related to each other, the first SNP suggests a recent point mutation (non-synonymous in this case) while the second SNP is likely to be introduced by recombination. The latter two analyses require a phylogenetic framework.

Homologous recombination in bacteria, which typically takes the form of gene conversion, facilitates adaptive evolution in bacteria [47,48]. Indeed, gene conversion underlines much of the hypervariability observed at loci encoding dominant antigens such as *ospC* in LB-group *Borrelia* and the *rfb* operon (coding for the O-antigen) and the *fim* locus (coding for an adhesion) in *Escherichia coli*[17,49]. We have previously established that geographically coexisting *Borrelia* strains recombine at a rate approximately three times the mutation rate [17,25]. Identification of homoplasy, a genetic variation that is inconsistent with the overall genome phylogeny, forms the basis of many tests of recombination such as the four-gamete test and the homoplasy test [50-52]. With sequence alignments displayed in the context of a genome phylogeny, a user of *BorreliaBase* can visually identify genomic regions affected by recombination and even estimate local recombination rates based on identification of homoplasies. In the first example, among strains of the same *Borrelia* species (*B. burgdorferi* sensu stricto), the presence of all four haplotypes at a pair of SNP sites is indicative of at least one past recombination event involving one of the SNP sites (Figure 5A). The second example shows that a portion of the *b22* gene of the *B. burgdorferi* sensu stricto strain BOL26 is most similar to its homologs in *B. afzelii*, a different but sympatric species, than to its homologs in con-specific strains, indicating an unambiguous case of cross-species recombination (Figure 5B). Breakage points of this specific recombination event have been identified by recognizing where such phylogenetic inconsistency ends [17]. In the last example, one estimates recombination rates relative to mutation rates by scanning for sequence differences between a pair of sister-group strains (Figure 5C). A SNP segregating between two phylogenetic sister-group strains may be caused either by mutation or by recombination. For such a SNP site, if one of the two SNP states does not occur in any other strains in a large population sample it is likely to be a recent point mutation, while it is more likely to be introduced by recombination if it appears in other strains as well [53]. Since these visual methods for identifying footprints of recombination do not require performing any statistical analysis, in our experience *BorreliaBase* makes an effective pedagogical tool as well.

## Conclusions

The phylogeny-centered design of *BorreliaBase* encourages comparing *Borrelia* genomes in the framework of their shared phylogenetic history. More than simply serving as a graphic guide for selecting and de-selecting strains, the genome phylogeny provides visual, evolution-based expectations on comparative genomic information such as an average level of sequence variability and the expected phylogenetic consistency of SNPs. When, and only when, an observed pattern deviates significantly from these evolutionary expectations should functional importance be conjectured. For example, the observed high levels of within-species sequence variability of lipoprotein genes such as *ospC, dbpA*, and *vls* deviate greatly from the expected sequence variability, suggesting strong diversifying selection at these loci driven by, e.g., immune escape from the host. Conversely, the observed high levels of sequence conservation of *cis-*regulatory elements deviate from the expected level of sequence variations between *Borrelia* species, consistent with gene-regulatory roles these IGS elements play (Figure 4). A phylogenetic expectation is especially valuable for identifying genomic footprints of recombination (Figure 5). Nexplorer, an online tool for analyzing evolution of characters (e.g., presence and absence of introns) associated with a protein family, similarly employs a phylogeny-centered user-interface design [54].

While allowing interactive comparative exploration of *Borrelia* genomes, the current version of *BorreliaBase* (public release 1.0) is admittedly limited in genomic scope. For example, comparative features, including gene synteny and orthologs, are only available for the three universally present replicons. We plan to include results of other replicons in future releases of *BorreliaBase*.

## Availability and requirements

The *BorreliaBase* is publicly accessible online at http://BorreliaBase.org. The source code of the website is publicly available at http://sourceforge.net/projects/phylobrowse/.

## Abbreviations

IGS: Intergenic Spacer; ORF: Open Reading Frame; B. burgdorferi s.l.: *Borrelia burgdorferi* sensu lato; B. burgdorferi s.s.: *Borrelia burgdorferi* sensu stricto; LB: Lyme borreliosis; RF: Relapsing fever.

## Competing interests

The authors declare that they have no competing interests.

## Authors’ contributions

LD designed and implemented the web interface. PP and CM developed prototype websites for ORFs and IGSs respectively. PP matched GenBank Locus Tags to individual ORFs in the back-end genome database. PP and SA identified consensus start-codon positions for each orthologous ORF family. GR prepared figures and online documentation. DP configures and administrates the website. EM, CF, SE BL, SC, and WQ sequenced and annotated the majority of the genomes in the current *BorreliaBase*. SC identified plasmids in these genomes. WQ conceived of the project, developed the back-end database, and drafted the manuscript. All authors read and approved the final manuscript.
